# Human organoids and organ-on-chip systems for AI-guided therapeutic discovery against emerging and re-emerging infections

**DOI:** 10.3389/fphar.2026.1927806

**Published:** 2026-09-01

**Authors:** Sarbjeet Kaur Makkar, Simran Bhatia, Nhi Nguyen, Guoli Wei

**Affiliations:** 1 Department of Internal Medicine, Division of Hematology and Oncology, University of Michigan, Ann Arbor, MI, United States; 2 Rogel Cancer Center, University of Michigan, Ann Arbor, MI, United States; 3 Department of Oncology, Affiliated Hospital of Integrated Traditional Chinese and Western Medicine, Nanjing University of Chinese Medicine, Nanjing, Jiangsu, China; 4 Department of Oncology, Jiangsu Province Academy of Traditional Chinese Medicine, Nanjing, Jiangsu, China

**Keywords:** antimicrobial resistance, artificial intelligence, drug repurposing, host-directed therapy, human organoids, infectious disease pharmacology, organ-on-chip, pandemic preparedness

## Abstract

Emerging and re-emerging infections expose a persistent mismatch between the speed of pathogen evolution and the pace of therapeutic development. Conventional two-dimensional cultures are scalable but poorly reproduce tissue architecture, whereas animal models may not capture human-specific tropism, immunity, pharmacokinetics, or toxicity. Human organoids and organ-on-chip systems offer complementary solutions by combining organ-specific human cells with three-dimensional architecture, barrier function, perfusion, mechanical forces, and, increasingly, immune and microbial components. This review evaluates their use in antiviral, antibacterial, antiparasitic, and antifungal pharmacology, with emphasis on drug repurposing, host-directed therapy, RNA and nanomedicine approaches, pharmacokinetic-pharmacodynamic assessment, and tissue-specific safety. We further examine how artificial intelligence, high-content imaging, multi-omics, and computational screening can convert these models into iterative discovery platforms that prioritize targets, compounds, combinations, and biomarkers. Current limitations include incomplete immune and vascular complexity, developmental immaturity, matrix and protocol variability, high cost, biosafety constraints, and limited prospective clinical validation. We propose a pandemic-ready pipeline in which pathogen sequencing and AI-based prioritization are followed by organoid and chip validation, mechanistic omics, multi-organ exposure and toxicity testing, and biomarker-guided clinical translation. Used as complementary rather than universal replacement models, these technologies could reduce false-positive leads, improve dose relevance, and accelerate development of therapeutics that control pathogens while preserving human tissue function.

## 1 Introduction

Emerging and re-emerging infectious diseases are shaped by microbial evolution, zoonotic spillover, ecological disruption, climate change, urbanization, population mobility, and unequal public-health capacity. These drivers continually create new opportunities for transmission and cross-species adaptation, while global connectivity allows local outbreaks to become international emergencies. The consequences are not restricted to novel viruses: antimicrobial resistance, resurgent tuberculosis, expanding vector-borne disease, and invasive fungal infections are simultaneously narrowing therapeutic options (Jones et al., 2008; Morens and Fauci, 2013; Baker et al., 2022; Carlson et al., 2022; GBD, 2021 Antimicrobial Resistance Collaborators, 2024).

Therapeutic preparedness remains uneven across pathogen classes. Approved antivirals are concentrated in a limited number of infections; antibacterial discovery is slowed by biological and commercial barriers; antiparasitic regimens are frequently toxic, prolonged, or vulnerable to resistance; and systemic antifungal therapy relies on relatively few drug classes (Silver, 2011; Roemer and Krysan, 2014; Theuretzbacher et al., 2019; Adamson et al., 2021; Fisher et al., 2022; Wijnant et al., 2022; GBD, 2021 Antimicrobial Resistance Collaborators, 2024). During an outbreak, de novo drug development is rarely compatible with the clinical timeline, making repurposing and combination therapy attractive. Yet the COVID-19 experience showed that activity in permissive cell lines does not ensure clinically achievable exposure, tissue efficacy, or benefit in randomized trials (Guy et al., 2020; Li et al., 2023; von Delft et al., 2023).

These limitations have shifted attention from screening more compounds to improving the biological context in which candidates are evaluated. Infection is a tissue-level process involving differentiated target cells, epithelial and endothelial barriers, extracellular matrix, mechanical forces, resident and circulating immune cells, microbial communities, and organ-specific metabolism. A useful preclinical system must therefore evaluate not only pathogen inhibition but also host injury, barrier function, inflammatory responses, drug penetration, and toxicity. Human organoids and organ-on-chip systems are positioned between reductionist assays and whole-animal studies and can provide this missing human tissue context (Kim et al., 2020; Ingber, 2022; Loewa et al., 2023).

This narrative review examines how organoids, organ chips, and their integration with artificial intelligence can support pharmacological discovery against viral, bacterial, parasitic, and fungal threats. Rather than cataloguing every available model, we focus on the capabilities that influence translational decision-making: tissue tropism, therapeutic response, host-directed effects, dose relevance, safety, reproducibility, and rapid adaptation to emerging pathogens.

Literature was identified through targeted searches of PubMed and publisher databases, supplemented by reference-list screening, using combinations of “organoid,” “organ-on-chip,” “microphysiological system,” “infectious disease,” “antiviral,” “antibacterial,” “antifungal,” “antiparasitic,” “drug repurposing,” “artificial intelligence,” and “PK/PD.” Priority was given to peer-reviewed primary studies, authoritative reviews, and regulatory documents available through June 2026. Because this is a narrative rather than systematic review, the search was designed to capture representative advances, translational applications, and major limitations rather than provide an exhaustive evidence synthesis.

## 2 Why conventional preclinical models are insufficient

### 2.1 Two-dimensional cultures

Two-dimensional cultures remain indispensable for pathogen isolation, mechanistic studies, genetic perturbation, and high-throughput screening. They are inexpensive, experimentally tractable, and compatible with standardized virological, microbiological, imaging, and biochemical readouts. Their weakness is not lack of utility but lack of physiological context. Flattened monolayers generally fail to reproduce apical-basal polarity, three-dimensional extracellular matrix, mucus, gradients, tissue-specific differentiation, stromal signaling, perfusion, and multicellular organization (Pampaloni et al., 2007; Barrila et al., 2010; Duval et al., 2017).

These omissions can change receptor expression, innate immune competence, metabolism, and drug response. A compound may suppress replication in an immortalized cell line only at concentrations that cannot be achieved safely in patients, while a potentially useful therapy may be missed because the model lacks the relevant differentiated cell type or host pathway. Monocultures are also poorly suited to evaluate host-directed agents whose benefit depends on epithelial repair, endothelial protection, immune-cell recruitment, or control of inflammatory injury (Edmondson et al., 2014; Langhans, 2018).

### 2.2 Animal models

Animal models provide systemic immunity, pathology, pharmacokinetics, and survival endpoints, and they remain essential for questions that cannot yet be answered *in vitro*. However, species differences in pathogen receptors, restriction factors, immune signaling, microbiomes, metabolism, and tissue distribution can limit translation. Human-adapted pathogens may require genetic modification of the host, pathogen adaptation, or immunosuppression, each of which can alter the biology being modeled (van der Worp et al., 2010; Johansen et al., 2020; Muñoz-Fontela et al., 2020).

Differences in inflammatory programs and toxicology are particularly relevant to anti-infective therapy, where therapeutic windows can be narrow and tissue exposure is often decisive. Animal studies also face ethical, cost, containment, and scalability constraints, especially for high-consequence pathogens and non-human primates (Olson et al., 2000; Seok et al., 2013; Golding et al., 2018; Jameson and Masopust, 2018). The appropriate goal is therefore not wholesale replacement of animal models, but a layered strategy in which human tissue systems remove weak candidates and clarify mechanisms before focused *in vivo* studies.

### 2.3 Consequences for drug discovery

Poor model predictability contributes to false-positive efficacy signals, missed toxicity, late-stage attrition, and inefficient outbreak response. Lack of efficacy and safety remain major reasons for clinical failure, while the cost of advancing unsuccessful candidates is amplified by long development timelines (Kola and Landis, 2004; Paul et al., 2010; Waring et al., 2015). Human-relevant models may improve candidate selection and reduce unnecessary animal and clinical testing, although their economic and predictive value must be demonstrated prospectively (Franzen et al., 2019; Loewa et al., 2023).

## 3 Human organoids and organ-on-chip systems

### 3.1 Definitions and complementary capabilities

Organoids are three-dimensional, self-organizing structures generated from adult tissue stem or progenitor cells, embryonic stem cells, or induced pluripotent stem cells. Adult stem cell-derived organoids often preserve mature epithelial programs and patient-specific features, whereas pluripotent stem cell-derived systems can model development and generate otherwise inaccessible tissues. Patient-derived organoids add genetic and phenotypic diversity that is valuable for precision pharmacology (Sato et al., 2009; Spence et al., 2011; Lancaster et al., 2013; Kim et al., 2020).

Organoids reproduce selected features of native tissue, including lineage diversity, polarity, lumen formation, barrier organization, and organ-specific function. They are not miniature complete organs: most lack full vasculature, circulating immunity, innervation, endocrine regulation, and microbiota unless these are deliberately introduced. Their value lies in providing a controllable human tissue context that is more physiological than a monolayer while remaining compatible with imaging, omics, gene editing, and drug screening (Kim et al., 2020; Blutt and Estes, 2022).

**FIGURE 1:**
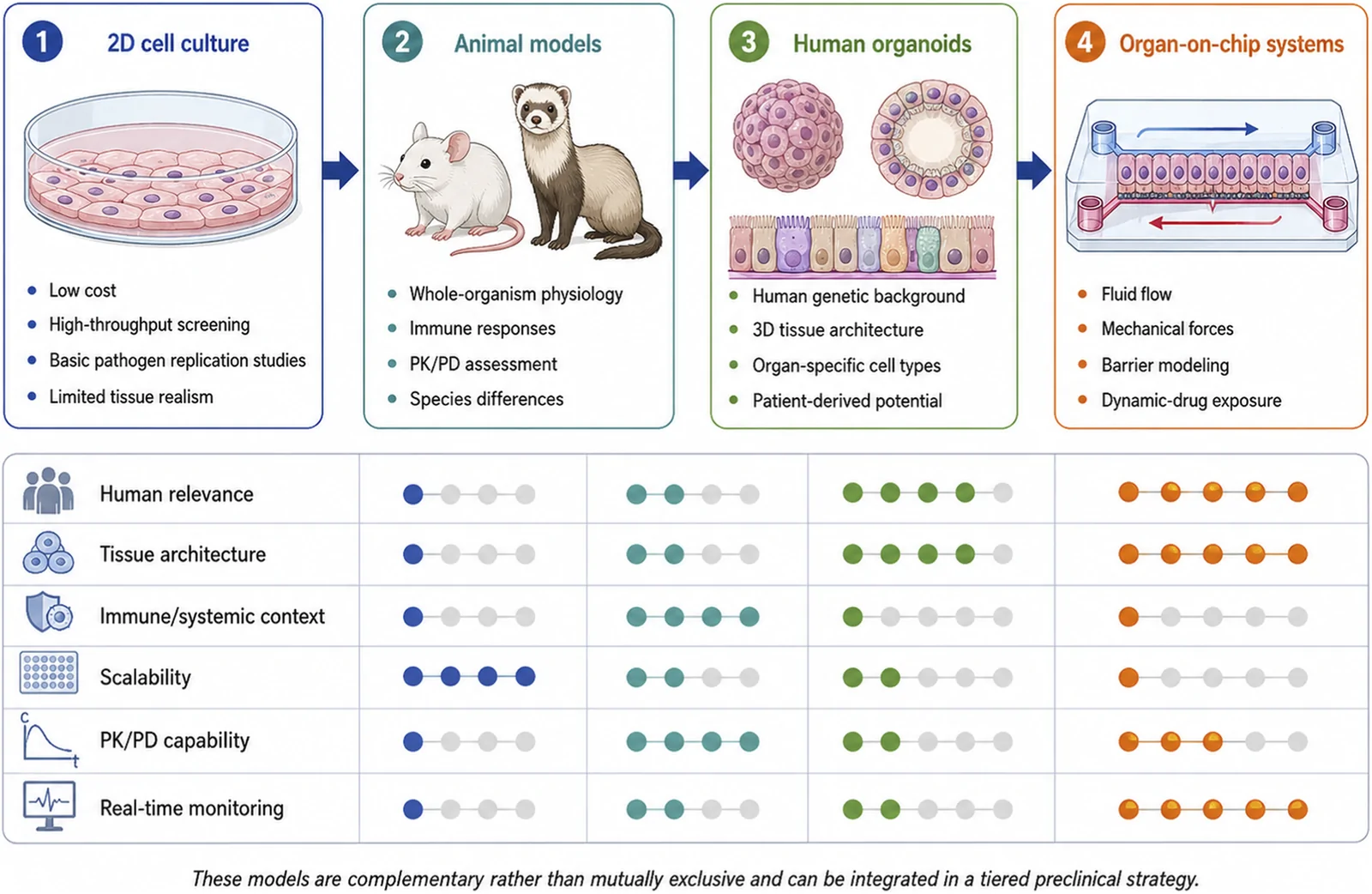
Comparison of preclinical models used in infectious disease drug discovery. The figure compares two-dimensional cell cultures, animal models, human organoids, and organ-on-chip systems. Each platform offers different strengths in human relevance, tissue architecture, immune or systemic context, scalability, PK/PD analysis, and real-time monitoring. Organoids and organ chips provide complementary human-relevant platforms and are best used alongside conventional cell and animal models. Abbreviations: 2D, two-dimensional; PK/PD, pharmacokinetics/pharmacodynamics.

Organ-on-chip systems are microengineered devices that culture living human cells under controlled flow, mechanical forces, and tissue-tissue interfaces. Microfluidic perfusion can reproduce vascular exposure and time-varying drug concentrations; flexible membranes can mimic breathing or peristalsis; parallel channels can model epithelial-endothelial barriers; and circulating immune cells can be introduced to study trafficking and inflammatory injury (Huh et al., 2010; Bhatia and Ingber, 2014; Ingber, 2022). Organoids and chips are therefore complementary: organoids provide self-organized cellular complexity, while chips provide physical control, access, and dynamic exposure. The complementary strengths and limitations of these model classes are summarized in Figure 1.

### 3.2 Organ-specific infection models

Respiratory organoids reproduce basal, secretory, ciliated, bronchiolar, or alveolar phenotypes and have been used to study SARS-CoV-2, influenza, respiratory syncytial virus, and other respiratory pathogens. They support analysis of entry-factor distribution, replication, interferon responses, ciliary injury, mucus changes, and antiviral activity in differentiated epithelium (Sachs et al., 2019; Han et al., 2021; Ekanger et al., 2022; Rajan et al., 2022). Lung and airway chips extend these capabilities by incorporating air-liquid interfaces, pulmonary endothelium, mechanical breathing motion, and immune recruitment. The human airway chip has been used to compare antiviral candidates under clinically relevant exposure conditions, illustrating its potential to distinguish tissue-active drugs from cell-line artifacts (Huh et al., 2010; Si et al., 2021).

Intestinal organoids are among the most mature infection models. They enabled cultivation of human rotavirus and norovirus, demonstrated productive SARS-CoV-2 infection of enterocytes, and supported studies of Salmonella invasion and Clostridioides difficile toxin-mediated barrier damage (Finkbeiner et al., 2012; Forbester et al., 2015; Leslie et al., 2015; Ettayebi et al., 2016; Lamers et al., 2020). Gut chips add luminal flow, peristalsis-like strain, mucus, oxygen gradients, and microbial co-culture. These features are particularly useful for studying pathogen-microbiome competition, dysbiosis, epithelial permeability, oral drug exposure, and gastrointestinal toxicity (Kim et al., 2012; Kim et al., 2016; Grassart et al., 2019; Jalili-Firoozinezhad et al., 2019).

Liver organoids and chips connect infection biology with metabolism and safety. Adult and pluripotent stem cell-derived liver tissues have been adapted to hepatitis B virus and malaria liver-stage infection, including human Plasmodium falciparum and P. vivax development (Huch et al., 2015; De Crignis et al., 2021; Yang et al., 2023; Nitaramorn et al., 2024). Perfused liver models can sustain hepatocyte function, support hepatitis B infection, and evaluate drug-induced liver injury, making them valuable for antiviral and antiparasitic candidates whose efficacy must be balanced against hepatic toxicity (Kang et al., 2017; Ortega-Prieto et al., 2018; Ewart et al., 2022).

Brain organoids provide access to human neurodevelopmental tissue and have clarified the vulnerability of neural progenitors to Zika virus and Japanese encephalitis virus. They have also been used to explore SARS-CoV-2-associated neural injury and candidate neuroprotective interventions, although direct neuroinvasion in patients remains debated (Garcez et al., 2016; Zhang et al., 2018; Mesci et al., 2022). Blood-brain barrier and brain chips complement these models by incorporating endothelial, pericyte, astrocytic, and neuronal compartments and by allowing quantitative assessment of barrier disruption and drug penetration (Wevers et al., 2018; Ahn et al., 2020; Boghdeh et al., 2022; Pediaditakis et al., 2022).

**FIGURE 2:**
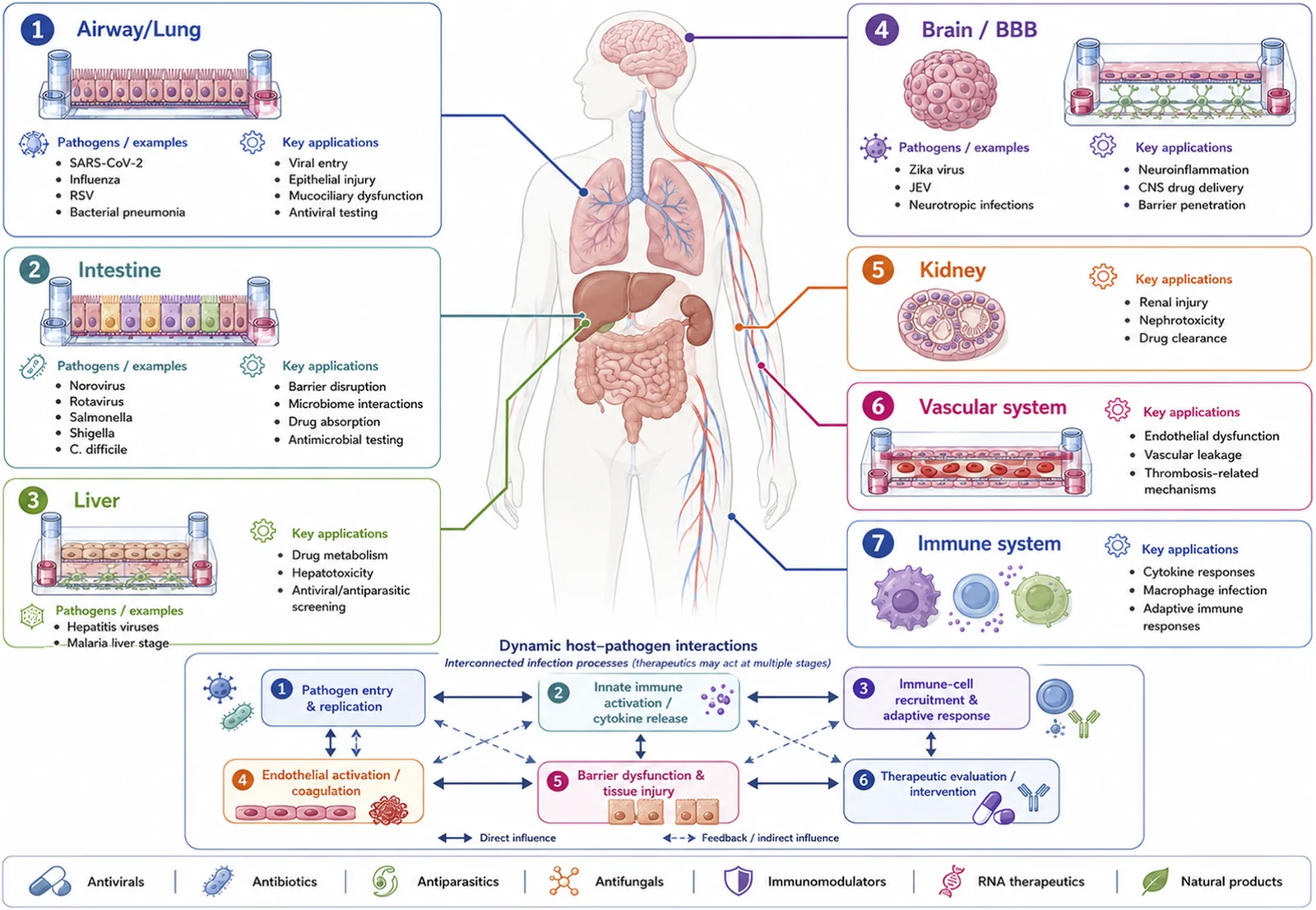
Representative organ-specific applications of human organoids and organ-on-chip platforms. Representative examples of airway, intestinal, liver, brain, kidney, vascular, and immune models are shown together with the pathogens and pharmacological questions they can address. The systems illustrated are intended as examples and are not exhaustive; organoid and organ-on-chip platforms can be developed for numerous additional tissues and organ systems depending on pathogen tropism, disease context, and the specific experimental or therapeutic question. These platforms can be used to evaluate pathogen entry and replication, host–pathogen interactions, barrier and tissue injury, therapeutic efficacy, drug transport, and tissue-specific toxicity across antiviral, antibacterial, antiparasitic, antifungal, immunomodulatory, RNA-based, and natural-product therapies. Abbreviations: BBB, blood–brain barrier; CNS, central nervous system; JEV, Japanese encephalitis virus; RSV, respiratory syncytial virus; SARS-CoV-2, severe acute respiratory syndrome coronavirus 2.

Kidney and vascular organoids are relevant to systemic infection, endothelial injury, thrombosis, and nephrotoxicity. SARS-CoV-2 studies showed infection and fibrotic responses in kidney organoids and therapeutic inhibition in engineered vascular and renal tissues (Takasato et al., 2015; Monteil et al., 2020; Jansen et al., 2022). Blood-vessel organoids and vascular-immune models permit analysis of endothelial permeability, complement, inflammatory macrophages, and candidate tissue-protective strategies (Wimmer et al., 2019; Kawakami et al., 2023; Chau et al., 2024). Tonsil and other immune organoids further provide germinal-center-like and antibody-response readouts, offering a route toward human vaccine and immunomodulator testing (Bar-Ephraim et al., 2020; Wagar et al., 2021). Representative organ-specific platforms and their principal pharmacological applications are illustrated in Figure 2.

### 3.3 Immune-integrated organ-on-chip and organoid systems

A major recent advance is the deliberate integration of immune components into organ-on-chip and organoid platforms. Depending on the infection and tissue, these systems can incorporate resident or monocyte-derived macrophages, neutrophils, dendritic cells, lymphocytes, or vascular immune compartments. This enables concurrent measurement of pathogen replication, leukocyte adhesion and transmigration, phagocytosis, cytokine release, endothelial activation, vascular leakage, and tissue injury rather than treating the immune response as a separate downstream event. Infection-focused organ-on-chip studies have already demonstrated the value of macrophage-containing lung and liver models, while vascular immune organoids have shown that SARS-CoV-2 can activate inflammatory macrophage programs linked to interferon signaling and tissue damage (Siwczak et al., 2022; Chau et al., 2024; Alonso-Roman et al., 2024).

Immune complexity should nevertheless be matched to the pharmacological question. Neutrophils and macrophages are particularly relevant to acute bacterial and fungal infection, whereas dendritic cells and T- and B-cell components become important when antigen presentation, adaptive immunity, vaccine responses, immune exhaustion, or immunomodulatory therapy are being studied. Immune-cell source, activation state, donor background, residence time, and compatibility with perfusion conditions can substantially influence readouts. Thus, immune-integrated chips are most informative when the immune compartment is selected prospectively and validated with functional endpoints rather than added solely to increase model complexity (Bar-Ephraim et al., 2020; Wagar et al., 2021; Alonso-Roman et al., 2024).

### 3.4 Genetically engineered organoids and organ-on-chip models

Genetic engineering adds a causal layer to human tissue models by allowing individual host factors to be perturbed in an otherwise matched cellular background. CRISPR-Cas9 knockout, knock-in, base-editing, or transcriptional perturbation can be introduced before organoid differentiation or into expandable adult stem-cell organoids, after which edited tissues can be incorporated into microfluidic devices. Isogenic pairs are particularly valuable for separating the effect of a candidate susceptibility gene from donor-to-donor variation and for validating targets identified by computational, genomic, or CRISPR screens. In coronavirus research, a CRISPR-engineered intestinal organoid biobank confirmed ACE2 and DPP4 receptor dependence and identified TMPRSS2 as a critical protease for coronavirus entry, demonstrating how edited organoids can test whether host factors identified in simpler cell systems remain relevant in non-transformed human tissue (Beumer et al., 2021). A multi-organoid study subsequently combined organ-specific infection models with CRISPR validation to identify CIART and other host-response genes that influence SARS-CoV-2 infection (Tang et al., 2023).

These approaches are well suited to target validation, host-directed drug discovery, genotype-phenotype studies, and modeling of inherited susceptibility. Their limitations include off-target editing, clonal selection, altered differentiation, mosaicism, and the possibility that a genetic knockout produces a phenotype that does not reproduce partial pharmacological inhibition. For this reason, genetic perturbation should ideally be paired with rescue experiments, orthogonal editing strategies, pharmacological validation, and appropriate isogenic controls.

### 3.5 Strengths, limitations, and model selection

No platform is universally superior. Organoid choice should be guided by the biological question, target tissue, required maturity, and relevant host compartments. Chips are most valuable when flow, barriers, mechanics, or dynamic exposure are central; organoids are advantageous when lineage diversity, self-organization, or patient specificity is required. Hybrid organoid-on-chip systems can combine both strengths, but increased complexity must produce measurable predictive gain rather than technological sophistication alone.

An infection-specific constraint is the orientation and accessibility of the epithelial surface. Many organoids are apical-in and therefore require microinjection, mechanical opening, polarity reversal, organoid-derived monolayers, or transfer to a chip to expose the relevant surface. These approaches are not biologically interchangeable and can alter differentiation, barrier properties, effective inoculum, matrix diffusion, and measured drug response. Infection route, matrix composition, accessible target-cell number, and normalization strategy should therefore be reported explicitly (Puschhof et al., 2021; Blutt and Estes, 2022).

The maturity of the evidence is also uneven across pathogen classes. Airway and intestinal viral models are comparatively well developed, whereas many bacterial, fungal, parasitic, immune, and multi-organ applications remain at proof-of-concept or early validation stages, with limited prospective benchmarking against clinical efficacy (Kim et al., 2020; Ingber, 2022; Loewa et al., 2023) (Table 1).

**TABLE 1:** Representative human organoid and organ-on-chip models for infectious disease pharmacology.

Platform	Representative pathogens/applications	Key pharmacological readouts	Principal limitations
Airway/lung organoids and chips	SARS-CoV-2, influenza, RSV, bacterial pneumonia	Entry and replication, ciliary injury, cytokines, barrier integrity, antiviral and anti-inflammatory response	Incomplete systemic immunity; variable alveolar maturity
Intestinal organoids and gut chips	Norovirus, rotavirus, Salmonella, Shigella, C. difficile	Epithelial invasion, toxin injury, permeability, microbiome interaction, oral drug effects	Closed lumens or unstable microbial co-culture
Liver organoids and liver chips	HBV, malaria liver stages, systemic inflammation	Pathogen development, drug metabolism, hepatotoxicity, stage-specific screening	Variable hepatocyte maturity and zonation
Brain organoids and BBB chips	Zika, Japanese encephalitis, neurotropic viruses and fungi	Neurotropism, developmental injury, BBB penetration, neurotoxicity	Limited vascular, immune, and adult neural maturity
Kidney/vascular/immune models	Viral kidney injury, endotheliopathy, sepsis, immune activation	Nephrotoxicity, vascular leakage, complement, immune recruitment, cytokines	Incomplete whole-body regulation and coagulation
Multi-organ chips	Systemic infection and combination therapy	PK/PD, metabolism, inter-organ toxicity, target-tissue exposure	Scaling, medium compatibility, device complexity

## 4 Applications across pathogen classes

### 4.1 Viral infections and antiviral discovery

Viruses depend on host receptors, proteases, trafficking machinery, metabolism, and innate immune state; consequently, viral susceptibility can vary sharply across cell types and tissues. Organoids and chips help define tissue tropism and distinguish entry from productive replication and tissue injury. For SARS-CoV-2, human airway, intestinal, kidney, and vascular systems demonstrated organ-specific infection and the importance of ACE2- and protease-dependent entry (Hoffmann et al., 2020; Lamers et al., 2020; Monteil et al., 2020). Similar approaches can be adapted rapidly to newly sequenced pathogens or variants.

These models are also useful for direct-acting antiviral screening. Polymerase, protease, entry, fusion, assembly, and release inhibitors can be tested in the tissue in which therapeutic exposure is required. Lung and colonic organoids identified SARS-CoV-2 inhibitors, while airway chips evaluated influenza and coronavirus candidates under dynamic and clinically plausible conditions (Han et al., 2021; Si et al., 2021). Human tissue models can additionally measure epithelial repair, barrier function, cytokine responses, and neurotoxicity, thereby extending antiviral discovery beyond simple reduction in viral RNA (De Clercq and Li, 2016; Mesci et al., 2022).

### 4.2 Bacterial infections, antimicrobial resistance, and tuberculosis

Organoids and chips can model bacterial attachment, epithelial invasion, intracellular survival, toxin production, biofilm-related tolerance, and tissue damage. Intestinal organoids reproduce Salmonella invasion and C. difficile toxin injury, while intestine chips show how architecture and mechanics influence Shigella infection (Forbester et al., 2015; Leslie et al., 2015; Grassart et al., 2019). Lung and liver chips have modeled viral-bacterial coinfection, endothelial damage, macrophage polarization, and Staphylococcus aureus persistence (Deinhardt-Emmer et al., 2020; Siwczak et al., 2022).

This tissue context is especially important for antimicrobial resistance. Standard minimum inhibitory concentrations measure planktonic growth but do not capture mucus, biofilms, intracellular niches, immune pressure, or impaired perfusion. Models of MRSA, carbapenem-resistant Enterobacterales, Pseudomonas aeruginosa, and Acinetobacter baumannii should therefore evaluate bacterial burden together with epithelial or endothelial integrity, inflammation, and host toxicity. The urgency is reinforced by the global AMR burden and the limited innovation in antibacterial pipelines (Silver, 2011; Tacconelli et al., 2018; Theuretzbacher et al., 2019; GBD, 2021 Antimicrobial Resistance Collaborators, 2024).

Tuberculosis requires specialized models because Mycobacterium tuberculosis persists in macrophages and heterogeneous granulomatous lesions. Human granuloma-like systems reproduce aspects of dormancy and resuscitation, while lung chips reveal contributions of alveolar epithelium to early bacterial control (Guirado and Schlesinger, 2013; Kapoor et al., 2013; Thacker et al., 2020). Drug penetration into macrophages, hypoxic regions, and caseous material can be as important as intrinsic potency; lesion-relevant binding and penetration assays should therefore be coupled to organoid or chip models when prioritizing regimens (Sarathy et al., 2017).

### 4.3 Parasitic and fungal infections

Parasites present stage- and tissue-specific vulnerabilities. Liver organoids support malaria pre-erythrocytic development and create opportunities to screen prophylactic, liver-stage, and potentially hypnozoite-directed therapies while simultaneously assessing hepatotoxicity (Yang et al., 2023; Nitaramorn et al., 2024). This need is heightened by emerging artemisinin resistance (Balikagala et al., 2021). Macrophage-based models are central to leishmaniasis because clinically relevant amastigotes reside in phagolysosomal compartments; human stem-cell-derived macrophages can support standardized high-content screening (Wijnant et al., 2022; Baert et al., 2024). Trypanosomatid and toxoplasmosis programs similarly require models that capture chronic tissue reservoirs, intracellular exposure, and CNS penetration (Field et al., 2017; Dunay et al., 2018; De Rycker et al., 2023).

Fungal drug discovery is constrained by the eukaryotic similarity between fungi and humans, limited systemic drug classes, toxicity, and rising resistance (Roemer and Krysan, 2014; Fisher et al., 2022). Human tissue models can measure adhesion, yeast-hypha transitions, biofilm formation, epithelial invasion, vascular dissemination, and immune-mediated injury. Aspergillosis-on-chip models enable quantitative treatment studies, and brain organoids or barrier chips may help address cryptococcal neurotropism and CNS delivery (Lockhart et al., 2017; Hoang et al., 2022). These platforms are particularly relevant to Candida auris, invasive aspergillosis, and cryptococcosis in immunocompromised patients.

## 5 Therapeutic strategies enabled by human tissue models

### 5.1 Host-directed therapy and immunomodulation

Host-directed therapies target pathways required for pathogen entry or replication, strengthen protective immunity, or limit damaging inflammation and tissue remodeling. Potential targets include interferon and JAK-STAT signaling, NF-kB activation, autophagy, inflammasomes, cellular metabolism, oxidative stress, epithelial repair, vascular stability, and fibrosis (Deretic et al., 2013; Broz and Dixit, 2016; Zumla et al., 2016; Kaufmann et al., 2018). Their appeal is the possibility of broad-spectrum activity and a higher barrier to pathogen resistance; their risk is disruption of essential host defense.

Clinical experience demonstrates the importance of timing and disease stage. Dexamethasone, JAK inhibition, and IL-6 receptor blockade improved outcomes in selected patients with severe COVID-19, whereas interferon strategies are more likely to be effective early, before inflammatory injury predominates (Marconi et al., 2021; Monk et al., 2021; RECOVERY Collaborative Group, 2021; REMAP-CAP Investigators, 2021). In prolonged sepsis or chronic infection, immune exhaustion may create a different therapeutic need from early hyperinflammation (Hotchkiss et al., 2013). Organoids and immune-competent chips are valuable because they can measure pathogen burden and tissue protection simultaneously, identify when anti-inflammatory benefit becomes immunosuppressive, and define organ-specific therapeutic windows.

### 5.2 Drug repurposing and combination therapy

Repurposing can accelerate outbreak response because approved or clinically advanced drugs have existing safety, manufacturing, and pharmacokinetic information (Pushpakom et al., 2019; Guy et al., 2020). Computational network approaches, host-pathogen interaction maps, and CRISPR screens can identify actionable host targets and repositioning candidates (Gordon et al., 2020; Daniloski et al., 2021; Gysi et al., 2021; Smith et al., 2021). However, repurposing is vulnerable to false positives arising from transformed cell lines, non-physiological concentrations, and pharmacokinetic mismatch. Human organoids and chips provide a critical validation layer by testing candidates in relevant tissues at clinically plausible free-drug exposures.

Combination therapy can broaden coverage, suppress resistance, and pair direct pathogen control with tissue protection. Relevant combinations include antiviral agents with distinct targets, antibiotics with beta-lactamase or efflux inhibitors, antibiofilm compounds, antiparasitic stage-specific regimens, antifungal combinations, and direct antimicrobials combined with host-directed agents. Computational models can prioritize synergy, but infection microenvironment and host toxicity must be validated experimentally (Cokol et al., 2018; Stokes et al., 2020).

### 5.3 RNA therapeutics and nanomedicine

RNA therapeutics are programmable and therefore attractive for rapidly evolving pathogens. Small interfering RNAs and antisense oligonucleotides can suppress pathogen transcripts or host dependency factors; mRNA can encode vaccine antigens, antibodies, or immune modulators; and CRISPR-Cas13 systems can be programmed against RNA viruses (Crooke et al., 2018; Pardi et al., 2018; Freije et al., 2019; Setten et al., 2019; Abbott et al., 2020; Kulkarni et al., 2021). The dominant translational barrier is delivery to the correct tissue and intracellular compartment.

Lipid nanoparticles are the most clinically mature carriers for mRNA and siRNA, while polymeric nanoparticles, extracellular vesicles, and ligand-targeted systems offer alternative biodistribution and release properties (Blanco et al., 2015; Hou et al., 2021; Tenchov et al., 2021). Organoids and chips can quantify delivery efficiency, cell-type specificity, barrier penetration, endosomal escape, innate immune activation, dose-response relationships, and organ toxicity. Perfused airway, gut, liver, kidney, and BBB models are especially useful for evaluating route-specific delivery and nanoparticle transport (Kang et al., 2021).

### 5.4 Natural products and phytochemicals

Natural products remain a major source of antibacterial, antifungal, antiparasitic, and immunomodulatory scaffolds. Modern genome mining, metabolomics, molecular networking, synthetic biology, and AI-assisted dereplication have renewed access to plant-, microbial-, and marine-derived chemical diversity (Harvey et al., 2015; Newman and Cragg, 2016; Wright, 2017; Atanasov et al., 2021). Human tissue models can help distinguish true anti-infective activity from non-specific cytotoxicity and identify barrier-protective, antibiofilm, or host-directed effects that are missed in pathogen-only assays. Translation nevertheless requires chemical standardization, purity, reproducibility, mechanism-of-action studies, clinically realistic exposure, and assessment of drug interactions.

## 6 Artificial intelligence as an integrating layer

### 6.1 Target identification and compound prioritization

Artificial intelligence can integrate pathogen genomes, structural data, transcriptomics, proteomics, metabolomics, single-cell sequencing, CRISPR screens, clinical phenotypes, and host-pathogen interaction networks. These data can identify essential pathogen proteins, host dependency factors, tissue-specific response states, and biomarkers that would be difficult to prioritize manually (Vamathevan et al., 2019; Gordon et al., 2020; Daniloski et al., 2021). Virtual screening and molecular docking can rapidly rank approved drugs, investigational compounds, natural products, and novel chemical matter, but predicted binding must be followed by cellular and tissue validation (Pinzi and Rastelli, 2019; Maia et al., 2020).

Knowledge graphs and network medicine are particularly suited to repurposing because they connect drugs, targets, pathways, diseases, side effects, and clinical evidence. Deep learning can also identify unconventional antibacterial structures, as illustrated by discovery of halicin-like activity from chemical representations (Stokes et al., 2020; Gysi et al., 2021). These approaches are hypothesis generators rather than substitutes for pharmacology; incomplete datasets, publication bias, and poor representation of tissue context can produce confident but non-translatable predictions.

### 6.2 High-content phenotyping and combination prediction

Organoids and chips generate rich images that capture pathogen replication, cell death, barrier disruption, ciliary loss, organoid swelling, immune-cell recruitment, vascular leakage, fibrosis-like remodeling, and recovery. Image-based profiling converts these phenotypes into quantitative features, while machine learning can identify treatment-response signatures that are not obvious to human observers (Caicedo et al., 2017; Chandrasekaran et al., 2021; Lukonin et al., 2021; Moshkov et al., 2024). This is especially important for host-directed therapies, where preservation of tissue architecture may be as meaningful as reduction in pathogen load.

AI can also prioritize synergistic combinations and predict resistance from pathogen genomes, expression profiles, and chemical features. Such predictions must account for microenvironment, growth state, and tissue exposure; organoids and chips provide the experimental context needed to test whether computationally predicted synergy persists in human tissue and whether it introduces additive toxicity (Cokol et al., 2018; Kim et al., 2022).

The predictive value of AI is constrained by small and heterogeneous datasets, donor and batch effects, differences in imaging pipelines and device materials, class imbalance, and domain shift between laboratories. Models trained on a single platform may learn technical artifacts rather than transferable biology, while data leakage or non-independent training and test sets can inflate apparent performance. Harmonized metadata, external validation, uncertainty estimates, interpretable features, and prospective testing across independent donors and devices are therefore essential (Vamathevan et al., 2019; Chandrasekaran et al., 2021; Moshkov et al., 2024).

### 6.3 A closed-loop discovery model

**FIGURE 3:**
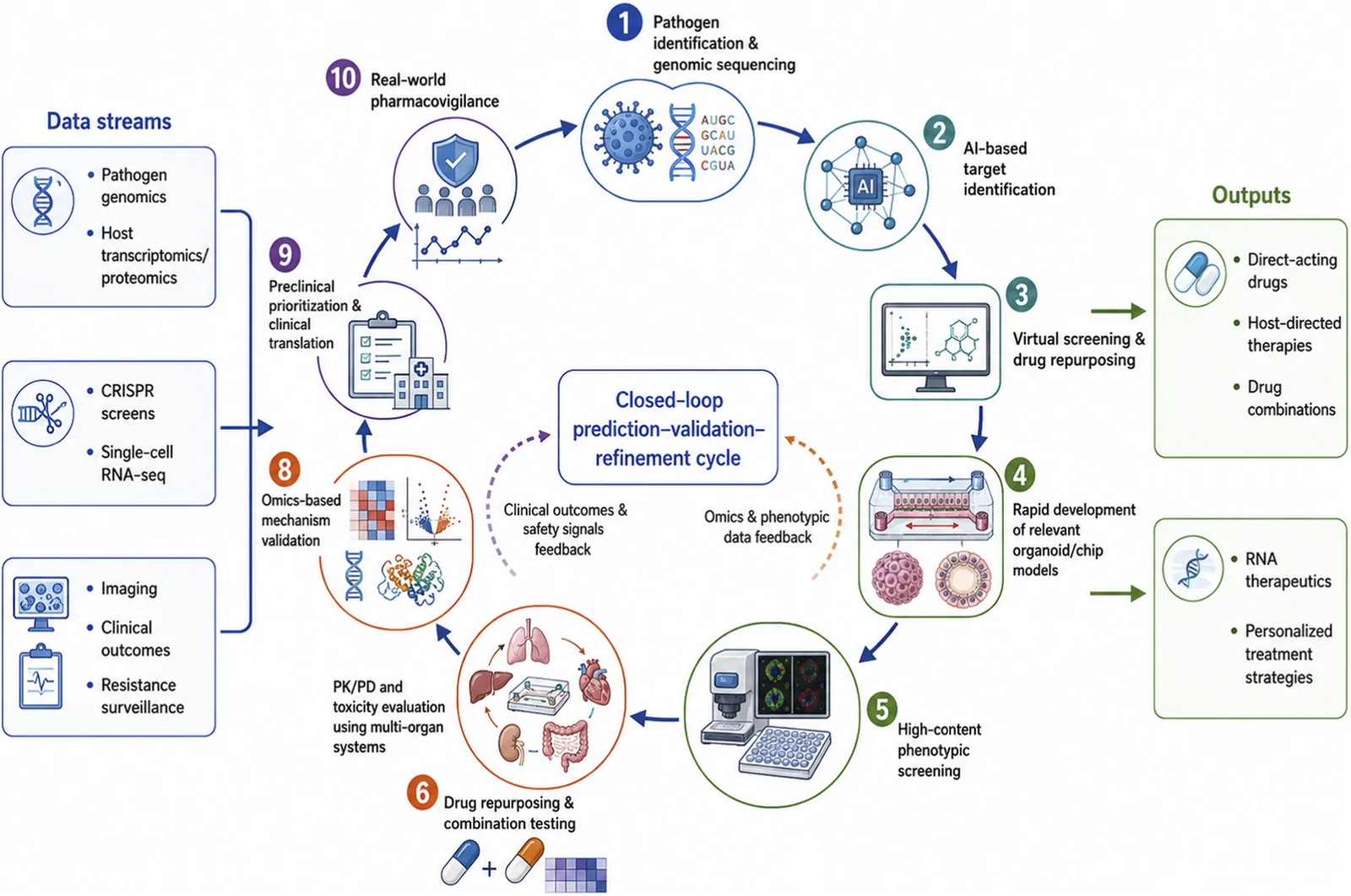
AI-guided, pandemic-ready therapeutic discovery pipeline. Pathogen sequencing and host-response data are integrated by artificial intelligence to prioritize targets, repurposed drugs, new compounds, and therapeutic combinations. Candidates are then tested in relevant organoid and organ-on-chip models using high-content imaging, omics, PK/PD, and toxicity analyses. Experimental and clinical data are returned to the computational models to refine subsequent predictions and support the development of direct-acting drugs, host-directed therapies, RNA therapeutics, combination regimens, and personalized treatments. Abbreviations: AI, artificial intelligence; CRISPR, clustered regularly interspaced short palindromic repeats; PK/PD, pharmacokinetics/pharmacodynamics; RNA-seq, RNA sequencing.

The strongest use of AI is an iterative closed loop. Computational models nominate targets and compounds; organoids and chips test efficacy, toxicity, and tissue specificity; imaging and omics define mechanisms and failure modes; and the resulting data refine the next prediction cycle. This design turns human tissue models from confirmatory endpoints into active data-generating components of discovery. This iterative prediction-validation-refinement workflow is shown in Figure 3.

### 6.4 Limitations and barriers to clinical implementation of AI-guided workflows

Although AI can accelerate prioritization and phenotypic analysis, several barriers currently limit clinical implementation. Training datasets from organoids and chips are often small, platform-specific, and affected by donor variability, batch effects, device materials, imaging settings, culture protocols, and differences in infection dose or timing. As a result, models may perform well within one laboratory but fail after transfer to another platform or patient population. Clinical deployment will require harmonized metadata and endpoints, independent external validation, explicit uncertainty estimates, interpretable outputs, and prospective testing in cohorts that represent biological and demographic diversity. Data leakage, class imbalance, publication bias, and overfitting are particularly important when sample numbers are modest (Vamathevan et al., 2019; Chandrasekaran et al., 2021; Moshkov et al., 2024).

Operational barriers are equally important. AI-guided organoid or chip workflows must be compatible with reproducible cell manufacturing, automated liquid handling and imaging, clinically meaningful turnaround times, secure and interoperable data infrastructure, and predefined quality-control criteria. Regulatory acceptance is likely to occur first in narrowly defined contexts of use rather than through a single general-purpose AI model. Prospective comparison with clinical exposure-response, efficacy, and safety outcomes will therefore be essential before AI-derived rankings can be used to support treatment selection or regulatory decision-making.

## 7 Precision pharmacology, PK/PD, and safety

### 7.1 Patient-specific susceptibility and response

Patient-derived organoids can preserve host genetic background, tissue-specific differentiation, and aspects of individual drug response. Evidence from oncology and cystic fibrosis demonstrates that *ex vivo* organoid responses can correlate with clinically meaningful treatment phenotypes (Dekkers et al., 2016; Vlachogiannis et al., 2018). Infectious disease applications are less mature but could be valuable in chronic, recurrent, drug-resistant, or immunocompromised settings, where there is time to generate cultures and compare regimens.

Host genetics can influence receptor expression, innate sensing, interferon responses, complement, macrophage activation, coagulation, and tissue repair. Rare defects in type I interferon immunity and neutralizing interferon autoantibodies helped explain severe COVID-19 in selected patients (Casanova, 2015; Bastard et al., 2020; Zhang et al., 2020). Patient-derived organoids combined with autologous immune cells and isogenic gene correction could link genotype to tissue susceptibility and identify pathway-specific interventions.

### 7.2 PK/PD and tissue penetration

Anti-infective efficacy depends on unbound drug exposure at the anatomical site of infection, not plasma concentration alone. Relevant PK/PD indices include time above the minimum inhibitory concentration, peak-to-MIC ratio, and exposure-to-MIC ratio, but the dominant index differs among drug classes (Mouton et al., 2005). Tissue barriers, protein binding, intracellular localization, granulomas, biofilms, mucus, and the BBB can decouple plasma and target-site exposure (Jager et al., 2019).

Microfluidic models can reproduce changing concentration-time profiles and allow serial measurement of parent drug, metabolites, pathogen burden, cytokines, and tissue injury. Fluidically coupled organ chips have demonstrated quantitative PK modeling, and multi-organ systems can link hepatic metabolism to renal, cardiac, neural, and target-organ toxicity (Herland et al., 2020; Ronaldson-Bouchard et al., 2022).

### 7.3 Therapeutic window and organ-specific toxicity

The therapeutic window should be defined using parallel efficacy and safety endpoints. Liver, kidney, cardiac, neural, vascular, and immune models can detect hepatotoxicity, nephrotoxicity, electrophysiological effects, neurotoxicity, endothelial injury, and immune suppression. Liver-chip studies illustrate the potential for improved prediction of drug-induced liver injury (Ewart et al., 2022). For combinations, tissue models can identify regimens that are microbiologically synergistic but pharmacologically unacceptable because of additive organ toxicity.

## 8 Biosafety, standardization, and regulatory translation

### 8.1 Biosafety and model hierarchy

High-risk pathogens require containment compatible with microfluidic tubing, pumps, sampling, imaging, and waste disposal. Pseudovirus, inactivated pathogen, surrogate, and reporter systems can support safer early-stage studies, particularly for entry and neutralization, but they do not fully reproduce replication, immune evasion, or authentic tissue injury. Pseudovirus results should therefore be validated with replication-competent organisms when the scientific question extends beyond entry (Nie et al., 2020; Cantoni et al., 2023).

### 8.2 Reproducibility and scalability

Variation arises from donor genetics, stem cell line, passage number, differentiation efficiency, organoid size, matrix composition, medium, infection route, multiplicity of infection, device geometry, flow rate, membrane properties, and endpoint selection. Undefined matrices such as Matrigel introduce lot-to-lot variability, motivating development of synthetic and chemically defined alternatives (Aisenbrey and Murphy, 2020). Reproducible studies should report cell source, maturity markers, culture conditions, inoculation method, exposure schedule, device parameters, and functional quality controls.

Scalable use requires plate-compatible formats, automated organoid formation, robotic liquid handling, standardized infection workflows, and high-content imaging. Microcavity arrays and related platforms demonstrate that organoid size and culture can be automated for screening (Brandenberg et al., 2020). For chips, reduced tubing, passive or integrated flow, mass-manufacturable materials, and minimized drug absorption are needed to move beyond bespoke prototypes (Low et al., 2021; Leung et al., 2022; Alver et al., 2024).

### 8.3 Regulatory acceptance

Most infectious-disease organoid and chip studies remain mechanistic or proof-of-concept demonstrations rather than prospectively qualified predictors of clinical efficacy. Liver toxicity and coupled PK applications have comparatively stronger benchmarking, but infection-specific contexts of use still require reference compounds, blinded testing, interlaboratory reproducibility, and direct comparison with clinical exposure-response data (Herland et al., 2020; Low et al., 2021; Ewart et al., 2022).

Regulatory adoption is therefore likely to proceed through clearly defined contexts of use rather than universal replacement of existing preclinical models. Validation should include human biological relevance, technical characterization, benchmark compounds, repeatability, sensitivity, specificity, robustness, and fit-for-purpose performance. Transparent AI pipelines, traceable cell sources, quality-controlled manufacturing, and predefined acceptance criteria will also be essential (Low et al., 2021; Leung et al., 2022).

Regulatory momentum is nevertheless increasing. The U.S. Food and Drug Administration’s 2025 roadmap explicitly identified organ-on-chip systems, computational modeling, and advanced *in vitro* assays as new approach methodologies that may reduce animal testing. Its 2026 draft guidance frames validation around a defined context of use, human biological relevance, technical characterization, and fit-for-purpose performance (U.S. Food and Drug Administration, 2025; U.S. Food and Drug Administration, 2026). These developments support stepwise qualification of human-relevant models while preserving evidentiary standards for safety and efficacy.

## 9 Future directions and a pandemic-ready discovery framework

### 9.1 Building more complete human models

Future models should incorporate immune cells according to the biological question rather than maximizing complexity indiscriminately. Macrophages and neutrophils are critical for bacterial and fungal disease; dendritic, T, and B cells are needed for antigen presentation and adaptive responses; and tissue-resident immunity is essential for mucosal infection. Immune organoids and tissue-immune co-cultures provide a route toward vaccine, adjuvant, and immunomodulator evaluation (Bar-Ephraim et al., 2020; Wagar et al., 2021).

Vascularization and perfusion can improve maturation, drug delivery, immune trafficking, and modeling of systemic dissemination. Engineered vascular-like brain organoids and flow-enhanced kidney organoids illustrate how physical perfusion can change tissue function (Cakir et al., 2019; Homan et al., 2019). Microbiome-integrated systems are equally important for gut, lung, oral, skin, and reproductive tract infections; standardized organoid-microbe co-culture and anaerobic gut chips provide early frameworks (Jalili-Firoozinezhad et al., 2019; Puschhof et al., 2021).

Recent platform development is moving toward integrated rather than isolated forms of complexity. Vascularized organoid-on-chip systems can establish perfusable endothelial networks around three-dimensional tissues, improving nutrient delivery, maturation, and access for circulating cells (Quintard et al., 2024). Coupled multi-organ systems extend this concept by linking target tissues with liver, kidney, cardiac, or barrier modules to examine metabolism, distribution, toxicity, and inter-organ signaling (Herland et al., 2020; Ronaldson-Bouchard et al., 2022; Zhao et al., 2024). Microbiome-integrated gut chips provide a complementary direction by maintaining host-microbial interactions under controlled oxygen and flow conditions (Jalili-Firoozinezhad et al., 2019). Together, these developments create more realistic settings for studying dissemination, inflammatory amplification, drug exposure, and off-target toxicity.

A parallel trend is increased scalability. Plate-compatible microphysiological systems, automated organoid production, robotic liquid handling, high-content microscopy, and machine-learning-based image analysis can shift these platforms from low-throughput demonstrations toward screening and iterative model training (Brandenberg et al., 2020; Moshkov et al., 2024; Alver et al., 2024). The key challenge is to preserve physiological information while simplifying hardware and workflows sufficiently for reproducible use across laboratories.

### 9.2 Proposed pandemic-ready pipeline

A rapid-response pipeline should be established before the next outbreak. First, pathogen identification and genomic sequencing define likely targets, resistance determinants, and tissue tropism. Second, AI integrates structural, genomic, and host-interaction data to prioritize direct-acting and host-directed candidates. Third, pre-established organoid and chip libraries are selected according to predicted target organs. Fourth, high-content screening measures pathogen burden, barrier function, inflammation, tissue morphology, and toxicity. Fifth, direct agents, repurposed drugs, and combinations are evaluated at clinically plausible exposures. Sixth, multi-organ systems assess metabolism, PK/PD, and organ toxicity. Seventh, omics and genetic perturbation validate mechanism and identify biomarkers. Finally, the strongest candidates advance to focused animal studies, adaptive clinical trials, and real-world pharmacovigilance (Table 2).

The framework is deliberately iterative: clinical and experimental data return to the computational models, improving subsequent target and dose predictions. Its success will depend on reference cell banks, standardized protocols, diverse donor representation, biosafety-compatible devices, and equitable access. Because emerging infections disproportionately affect low- and middle-income settings, capacity building, technology transfer, affordable materials, and local scientific leadership must be treated as core infrastructure rather than optional dissemination.

**TABLE 2:** Closed-loop pandemic-ready pharmacological discovery framework.

Discovery stage	Core inputs	Human-model function	Decision output
Pathogen characterization	Genome, structure, clinical phenotype	Select target tissues and infection conditions	Model and target selection
AI prioritization	Host-pathogen networks, omics, compound libraries	Generate ranked targets, drugs, and combinations	Testable candidate set
Tissue validation	Organoids and organ chips	Measure pathogen control, tissue injury, and host response	Efficacy and mechanism ranking
Exposure and safety	PK data, liver/kidney/cardiac/BBB modules	Test clinically plausible exposure and organ toxicity	Therapeutic window
Mechanistic refinement	Single-cell and bulk omics, imaging, CRISPR	Identify biomarkers, resistance, and toxicity signatures	Refined AI model and regimen
Translation	Focused animal studies, adaptive trials	Bridge model endpoints to clinical biomarkers	Clinical candidate and dosing strategy
Post-deployment learning	Pharmacovigilance and resistance surveillance	Update models with real-world outcomes	Continuous preparedness

## 10 Discussion and conclusion

Human organoids and organ-on-chip systems are reshaping infectious disease pharmacology by allowing therapeutic candidates to be evaluated within human-relevant tissue architecture, barriers, mechanical forces, and multicellular interactions. Unlike conventional screens that focus primarily on pathogen inhibition, these platforms can simultaneously measure pathogen burden, epithelial or endothelial injury, barrier integrity, inflammatory responses, drug metabolism, and organ-specific toxicity. Their most immediate value is therefore not to replace two-dimensional cultures or animal models universally, but to complement them by eliminating weak candidates earlier, clarifying human-specific mechanisms, and defining clinically relevant therapeutic windows before resource-intensive *in vivo* and clinical studies (Herland et al., 2020; Kim et al., 2020; Ewart et al., 2022; Ingber, 2022; Loewa et al., 2023).

The next stage of development should prioritize predictive performance over model complexity. Standardized cell sources, maturity criteria, infection conditions, exposure schedules, functional endpoints, and reporting practices will be required for reproducibility across laboratories. Prospective comparison with clinical pharmacokinetic, efficacy, and safety data is equally important for establishing clearly defined regulatory contexts of use. Incorporating immune, vascular, stromal, and microbial components should be guided by the biological question, while scalable formats, defined matrices, and robust manufacturing will be essential for broader deployment (Aisenbrey and Murphy, 2020; Brandenberg et al., 2020; Low et al., 2021; Leung et al., 2022; Ronaldson-Bouchard et al., 2022; Alver et al., 2024).

In the near term, the most consequential advances are likely to be practical rather than maximal increases in biological complexity: standardized donor and cell-bank resources, plate-compatible chips, defined matrices, integrated immune modules, automated high-content readouts, and benchmark datasets that allow direct comparison across laboratories. These developments could make organoid and organ-chip assays more useful for candidate triage, mechanism-of-action studies, PK/PD refinement, and safety assessment during outbreaks. In parallel, genetically engineered isogenic models should make it easier to validate host-dependency factors and connect human genetic variation with therapeutic response.

Over the longer term, vascularized organoid-on-chip systems, fluidically coupled multi-organ platforms, patient-matched immune and microbiome components, and AI-supported digital models may enable increasingly individualized simulations of infection and treatment. Major challenges will remain, including developmental immaturity, long-term stability, scaling between organs, universal culture-media compatibility, absorption of drugs by device materials, biosafety for high-consequence pathogens, cost, technical expertise, equitable access, and the need for prospective clinical validation. Progress should therefore be judged by reproducibility and predictive gain rather than by complexity alone, with regulatory qualification pursued for clearly defined contexts of use.

AI can strengthen this transition when it is used as an integrating layer rather than as a stand-alone substitute for experimental evidence. In a closed-loop workflow, computational models can prioritize targets, compounds, combinations, and biomarkers; organoids and chips can then generate human tissue-level efficacy and toxicity data that refine subsequent predictions. Establishing pre-characterized model libraries, biosafety-compatible workflows, interoperable datasets, and equitable access before outbreaks occur could make this approach a practical component of pandemic preparedness. With rigorous validation and appropriate model selection, these technologies may accelerate the development of therapies that not only suppress pathogens but also preserve tissue function and improve the likelihood of meaningful clinical benefit.
